# Best practices for cysteine analysis

**DOI:** 10.70401/fos.2025.0010

**Published:** 2025-12-31

**Authors:** Feroza K. Choudhury, Gina M. DeNicola

**Affiliations:** Department of Metabolism & Physiology, H. Lee Moffitt Cancer Center and Research Institute, Tampa, FL 33612, USA.

**Keywords:** Cysteine, glutathione, thiol analysis, redox homeostasis, LC-MS, derivatization, N-ethylmaleimide

## Abstract

Accurate measurement of cysteine and related thiol-containing metabolites is essential for understanding cellular redox regulation. However, the intrinsic reactivity and instability of cysteine present substantial analytical challenges. This review summarizes the biochemical context of cysteine and glutathione metabolism, emphasizing their dynamic redox equilibria and physiological relevance. We critically examine existing analytical approaches, including mass spectrometry-based, enzyme-coupled, and colorimetric methods, and discuss their respective strengths and limitations. Particular attention is given to sample preparation, derivatization strategies, and reagent selection, as these steps are crucial for preserving native thiol-disulfide status. Among various alkylating agents, N-ethylmaleimide is identified as the most reliable for thiol stabilization in liquid chromatography-mass spectrometry (LC-MS) workflows, while specific reagents such as monobromobimane or β-(4-hydroxyphenyl)ethyl iodoacetamide (HPE-IAM) are required for persulfide and polysulfide detection. The review also highlights the pitfalls of using indirect surrogates—such as glutathione or cystathionine levels—to infer cysteine availability, which can lead to significant misinterpretation of metabolic states. We conclude that direct LC-MS-based quantification of cysteine and glutathione, combined with careful derivatization and sample handling, remains the most reliable and accurate approach currently available for the assessment of thiol metabolism and redox homeostasis.

## 1. Introduction

Thiol-containing metabolites are crucial for maintaining cellular redox homeostasis, with glutathione and cysteine playing key roles. These molecules exist in dynamic equilibrium between their reduced and oxidized forms to maintain proper cellular redox balance^[1]^. Under normal physiological conditions, glutathione exists primarily in its reduced form (GSH) at approximately 1–10 mM, comprising approximately 98% of total cellular glutathione^[2,3]^. Cysteine concentration is maintained at approximately 100 μM in cells to minimize the toxicity associated with high cysteine accumulation, and while cystine concentration tends to be low in cancer cells, it varies greatly between cell types^[4]^.

In the presence of reactive oxygen species (ROS), glutathione and cysteine undergo oxidation to form dimeric structures through disulfide bond formation between sulfur atoms. Under physiological conditions, intracellular hydrogen peroxide concentrations are approximately 100 nM, where they participate in various signaling processes, primarily through the oxidation of cysteine residues in target proteins^[5]^. During oxidative stress, intracellular hydrogen peroxide concentrations can increase up to 10 μM, activating various antioxidant defense mechanisms^[5]^. The reactivity of ROS towards protein cysteine residues is highly dependent on local structural organization and the cellular environment, which ensures selectivity and specificity in ROS-mediated signaling^[6]^. While the peroxiredoxin system serves as the primary antioxidant defense, glutathione plays an important role when ROS levels are elevated^[7]^. To maintain GSH in its reduced form, oxidized glutathione (GSSG) is converted back to GSH through NADPH-dependent electron transfer reactions catalyzed by glutathione reductase.

The glutathione tripeptide is comprised of cysteine, glutamate and glycine. Consequently, cysteine availability is limiting for glutathione biosynthesis. Cells obtain cysteine through two primary pathways: *de novo* synthesis from serine and homocysteine via the transsulfuration pathway, or direct uptake of cystine followed by NADPH-dependent reduction to cysteine. *In vivo* tracing demonstrated that while *de novo* synthesis of cysteine is not universal across all tissues or genetically engineered mouse tumor models, direct uptake of cystine is a universal feature^[8]^. Intracellular and extracellular glutathione catabolism by CHAC1 and GGT1, respectively, releases amino acids and provides another source of cysteine under cysteine limiting conditions^[9,10]^.

In human blood, cystine concentrations range from 59–67 μM, while cysteine is less abundant at 9–12 μM^[11]^. Although free cysteine has a thiol group pKa of approximately 8.3, this can vary dramatically from 3.5–13 depending on the local microenvironment. At the physiological pH of the blood and cytosol (7.4), cysteine with a lower effective pKa exists predominantly in its de-protonated, reactive thiolate form^[12,13]^. Under neutral pH conditions and in the absence of reducing agents, cysteine readily oxidizes to cystine. However, within the highly reducing intracellular environment, characterized by high GSH/GSSG and NADPH/NADP^+^ ratios, cysteine is maintained in its reduced form^[14]^.

## 2. Analytical Challenges and Methods for Thiol Detection

The analysis of free thiol-containing metabolites presents significant technical challenges due to their inherent susceptibility to oxidation during sample extraction and processing. Glutathione exhibits greater stability than cysteine during extraction as a consequence of the higher pKa of its thiol group, which provides some protection against oxidation.

### 2.1 Mass spectrometry-based approaches

Mass spectrometry (MS)-based methods represent the most widely adopted approach for glutathione and cysteine detection. These techniques typically involve metabolite extraction from biological samples, followed by gas chromatography or liquid chromatography (LC) separation, followed by MS analysis. The critical step in analyzing redox status is quenching the sample to trap the thiol-disulfide in its native state during the metabolite extraction process. Two approaches are mainly applicable for quenching thiol-disulfide exchange: acid-based quenching or derivatization of free thiols with cell-permeable alkylating agents^[15]^. In the acid-based approach, efficient quenching requires protein denaturation to ensure enzyme deactivation and thiol accessibility for protonation. Trichloroacetic acid (10–20%) has been found to be most effective in denaturing proteins while limiting free thiol oxidation^[15]^. The drawback of this method is that quenching is reversible since the thiol groups are not permanently maintained in their reduced state, making them prone to oxidation if the pH is increased.

In the derivatization approach, thiol containing metabolites can be stabilized through covalent alkylation with reagents such as N-ethylmaleimide (NEM), iodoacetamide (IAM), and iodoacetic acid (IAA). The resulting derivatized products exhibit enhanced stability and are compatible with liquid chromatography-mass spectrometry (LC-MS) analysis. This approach is complemented by using high organic solvent concentrations like 80% methanol for protein denaturation. Alternatively, TCA-based protein precipitation can be combined with subsequent derivatization of the free thiols by suitable alkylating agents^[16]^. NEM has proven far more effective than IAA or IAM as an alkylating agent. Among maleimide-containing reagents, NEM demonstrates superior selectivity for thiol groups compared to (R)-(+)-N-(1-phenylethyl)maleimide, with optimal reaction conditions at pH 7 to minimize side reactions^[17]^. At pH 7, NEM derivatizes the small thiol 2-nitro-5-thiobenzoic acid (NTB) 85-fold faster than IAA and 20-fold faster than IAM ^[15]^. IAA and IAM have significant limitations. IAA reacts with amino and carboxyl groups, creating unexpected by-products that convolute thiol-containing metabolite detection^[18]^. IAA and IAM also react with the sulfur atom of methionine, demonstrating poor specificity for thiol groups^[19]^. In contrast, NEM exhibits specificity for thiol groups at concentrations of 1–20 mM below pH 7^[15]^. The negative charge of IAA renders it membrane impermeable and not suitable as an in vivo quenching agent, whereas IAM is membrane permeable and reacts faster with thiols than IAA^[20]^. Considering these factors, NEM is the superior alkylating agent for thiol-containing small molecules analysis and is widely applied^[21–24]^.

**Figure 1.**
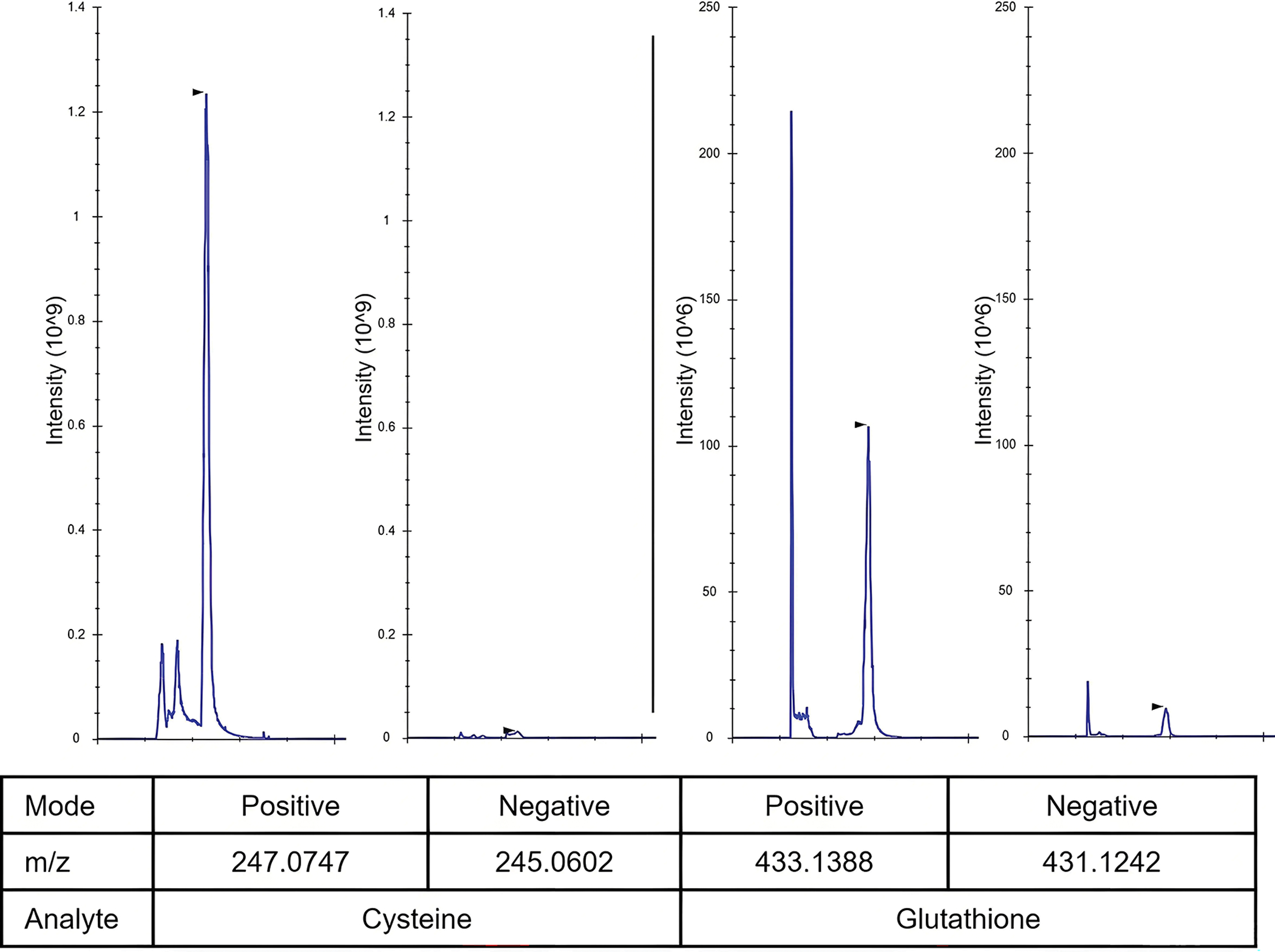
Chromatographic peaks of cysteine and glutathione were analyzed in both positive and negative ionization modes by mass spectrometry, demonstrating superior performance in positive mode. A 10 μM pure standard was derivatized with 5 mM NEM (pH 7) in 80% methanol and analyzed by LC-MS. Chromatography images were exported with Skyline. NEM: N-ethylmaleimide; LC-MS: liquid chromatography-mass spectrometry.

IAA and IAM produce carboxymethyl or carboxyamidomethyl derivatives, respectively, through irreversible reactions that yield highly stable adducts. NEM reacts with thiols via an addition reaction to produce thioether derivatives that remain stable when stored at cold temperatures (< 4 °C)^[22]^. However, NEM itself is unstable in alkaline conditions and may undergo hydrolysis to form N-ethylmaleimic acid, losing reactivity towards thiols. Furthermore, Cys-NEM can undergo intramolecular transamidation at pH > 9, and GSH-NEM can slowly regenerate the free thiol at pH 7–9, underscoring the importance of maintaining optimal pH^[15]^. NEM derivatives are best detected in positive ionization mode by mass spectrometer after chromatographic separation using hydrophilic interaction liquid chromatography (HILIC) methods (Figure 1).

### 2.2 Workflow for Thiol-Containing Metabolite Analysis with NEM Derivatization

**Figure 2.**
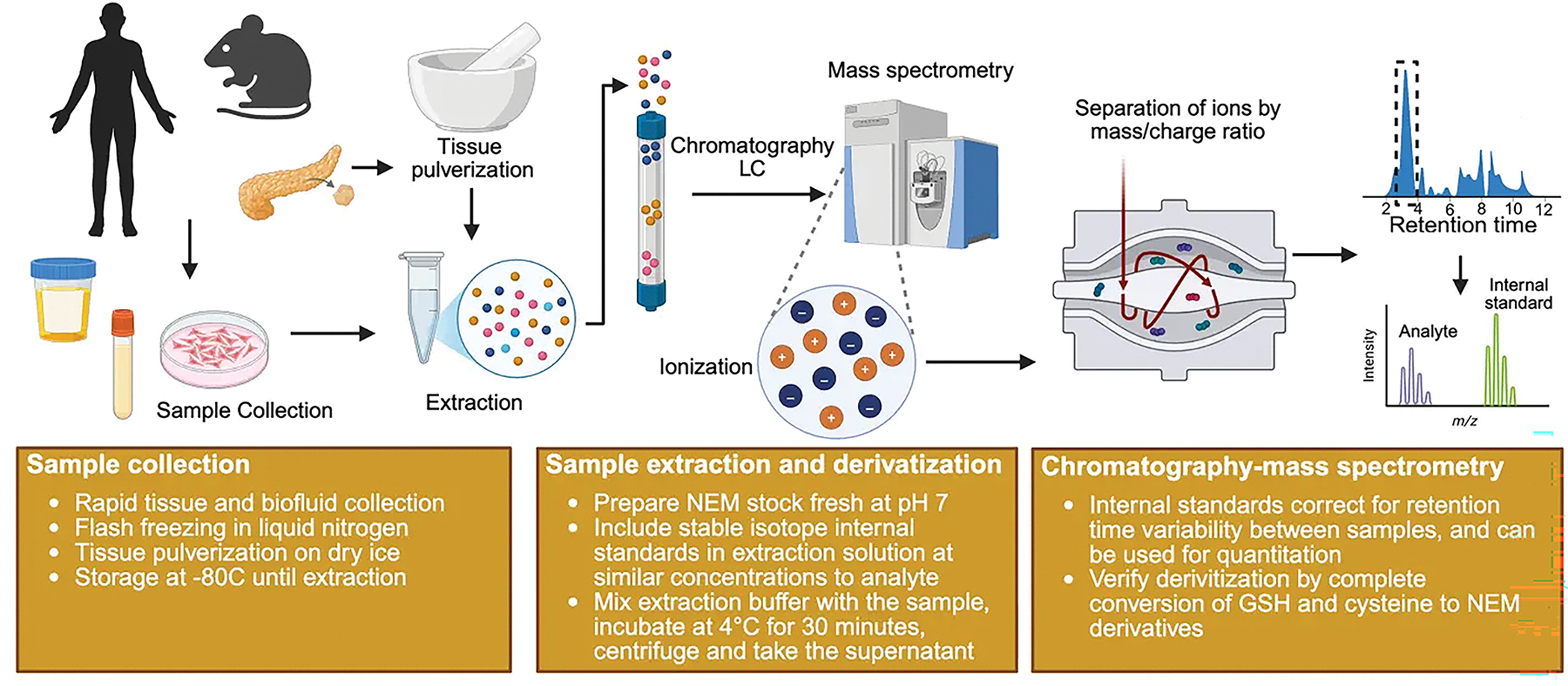
Workflow for NEM derivatization and analysis of thiol-containing metabolites. Created in Biorender.com. NEM: N-ethylmaleimide; LC: liquid chromatography.

This section describes an analytical workflow for derivatizing and detecting thiol-containing metabolites (Figure 2).

#### 2.2.1 Sample collection

Option 1 - Immediate freezing (preferred):

- Collect as quickly as possible and flash freeze in liquid nitrogen.
- Repeated freeze/thawing of the sample should be avoided.
- Store samples at −80 °C until analysis.

Option 2 - In-collection derivatization:

- Add NEM directly to biological fluids (plasma, saliva, urine) during collection to achieve a final concentration of 10 mM^[25]^.
- Centrifuge promptly after addition.
- This approach locks sulfhydryl groups and preserves their natural redox state^[25]^. Caution should be taken with the concentration of NEM included as it can impact the level of GSH detected in plasma^[25]^. RBCs contain a high concentration of GSH and may contribute to plasma GSH level via hemolysis.

#### 2.2.2 Reagent preparation

- Prepare 100 mM NEM stock solution: Add fresh NEM powder to 10 mM Ammonium formate solution (pH 7). Ensure pH is 7.0 to maintain the optimum pH during derivatization.
- For accurate quantification and retention time adjustments of metabolites, isotopically labeled internal standards for each target metabolite should be included in the extraction buffer. Stable isotope labeled version of thiol-containing metabolites (i.e. cysteine, glutathione) can be pre-reacted with 50 mM NEM (10 mM ammonium formate, pH = 7.0) solution for 30 minutes at room temperature to form a labeled NEM derivative, which can be used as an internal standard for that particular metabolite^[23,24]^.
- Prepare NEM extraction buffer: Add 100 mM NEM stock to methanol to make 5 mM NEM in 80% MeOH. Add the stable isotope labeled internal standard at a concentration similar to the target analyte.

#### 2.2.3 Sample extraction and derivatization

- Tissue samples: Grind tissue on dry ice to a fine powder. Add extraction buffer to a final concentration of 50 mg tissue/ml extraction buffer.
- Biological fluids: Thaw samples on ice. Add extraction buffer at a 1:10–1:20 dilution.
- Adherent Cells: Adherent cells at 70% confluency should be placed on ice and quickly washed with PBS. Add extraction buffer at the appropriate volume (e.g. 500 mL/well in a 6 well plate).
- Suspension cells: Mix cells with a large volume of cold PBS in a 50 mL conical and pellet. Add extraction buffer at 1million cells/ml concentration.

Once the samples are mixed with extraction buffer at the appropriate amount,

- Vortex the tissue, fluid, and suspension cells in extraction buffer. Rock the adherent cells in extraction buffer to ensure proper mixing.
- Incubate the samples for 30 minutes at 4 °C.
- For adherent cells, scrape cells and move the mixture into a 1.5 mL tube.
- Centrifuge the samples at 13,000 g for 10 minutes.
- Collect the supernatant containing the derivatized metabolites. Store at −80 °C until analysis or move it into LC-MS vials and analyze immediately.

#### 2.2.4 Analysis

Analyze the extract by LC-MS using HILIC chromatography in positive ionization mode.

#### 2.2.5 Method validation

While setting up the analytical method, it is important to validate that all cysteine and GSH are derivatized by NEM. Recovery rates should be measured by spiking non-derivatized isotopically labeled standard of known concentration in the biological matrix followed by extraction, derivatization, and analysis^[25,26]^. Intra-day and inter-day variability of the method should also be considered for robust analysis.

### 2.3 Other methods for thiol detection

There are times when NEM is not the optimal choice for thiol analysis. Persulfides are sulfur compounds structurally similar to disulfides with the formula of RSSH or RSS^−^, while polysulfides contain chains of covalently bonded sulfur atoms. Neither NEM nor IAA are suitable for detecting persulfides because the resulting persulfide adducts rapidly convert into their corresponding thioethers. For detecting persulfides, monobromobimane and *N*-*t*-butyl-iodoacetamide are more effective as the generated alkylated persulfide adducts are stable^[27]^. For the detection of polysulfide, tyrosine combined with a hydroxyphenyl moiety of HPE-IAM has a stabilizing effect and aids the detection of these reactive sulfur species^[28]^.

#### 2.3.1 Spatial analysis methods

Matrix-assisted laser desorption ionization-mass spectrometry (MALDI-MS) advancements have enabled the analysis of metabolism in spatial contexts. However, tissue sectioning disrupts organ structural homeostasis, exposing cysteine to rapid oxidation. The first reported derivatization reagent for MALDI-MS thiol detection is (E)-2-cyano-N-(2-(2,5-dioxo-2,5-dihydro-1H-pyrrol-1-yl)ethyl)-3-(4-hydroxyphenyl)acrylamide (CHC-Mal), which enables in situ derivatization for spatial distribution analysis of cysteine and glutathione^[29]^. For spatial analysis of archival tissue sections, reducing agents such as tris (2-carboxyethyl) phosphine hydrochloride (TCEP) or 2,5-dihydroxybenzoic acid (DHB) can increase detectable thiol content. However, since oxidized thiols cannot revert to their original redox state, data interpretation requires cautious interpretation.

#### 2.3.2 Enzyme-based detection methods

Several commercial enzyme-based assays are available for glutathione detection, including the Promega GSH/GSSG-Glo assay system. These kits utilize glutathione-S-transferase-mediated activation of glutathione probes in the presence of sample glutathione. Differentiation between oxidized and reduced glutathione is achieved through selective extraction buffers. Total glutathione is reduced to GSH for total detection, while for GSSG detection, GSH is chemically blocked to enable specific GSSG quantification after its reduction to GSH. A commercial kit is also available for cysteine detection from Abcam, based on the principle that cysteine reduces phosphotungstic acid to produce tungsten blue, which has an absorption peak at 600 nm. No such kit is currently available for cystine detection.

Ellman’s reagent (5,5’-dithiobis-2-nitrobenzoic acid, DTNB) provides a colorimetric method for free thiol detection, producing yellow NTB with absorbance at 412 nm^[30]^. However, this method lacks specificity for cysteine and detects all thiols groups present. Its utility is therefore limited to in vitro applications containing only a single thiol species^[31]^. Several derivatives of Ellman’s reagent have been developed for use under different conditions, such as 5-(2-Aminoethyl)dithio-2-nitrobenzoate (ADNB), which is suitable for alkaline conditions^[32]^.

## 3. Analytical Considerations and Recommendations

Researchers frequently use glutathione measurements as surrogates for cysteine availability to avoid the technical challenges of cysteine derivatization and mass spectrometry. However, these indirect approaches are fundamentally flawed and can lead to misinterpretation of cysteine metabolism.

### 3.1 Glutathione as a cysteine surrogate

Cellular redox status is commonly assessed through GSH/GSSG ratios, and total glutathione levels are sometimes used as a proxy for cysteine availability. However, this approach is fundamentally flawed for several reasons. First, cysteine levels significantly influence glutathione synthesis and degradation, but glutathione levels do not always reflect cysteine availability. Glutathione levels may decrease due to oxidation to GSSG, CHACl-mediated degradation to provide precursor amino acids, or impaired synthesis unrelated to cysteine availability. For example, insufficient glutamate or glycine, or decreased expression of glutathione-synthesizing enzyme glutamate-cysteine ligase catalytic subunit (GCLC), can all reduce glutathione levels independent of cysteine status.

Second, measuring GSH/GSSG ratios without proper derivatization is inherently inaccurate. Without alkylation or dedicated extraction buffers that lock the redox state, free thiols are prone to oxidation during extraction, artificially elevating GSSG levels and skewing the measured ratio.

### 3.2 Other indirect approaches

Researchers studying cysteine synthesis sometimes use cystathionine as a surrogate marker for cysteine production due to its stability and ease of measurement. However, cystathionine accumulation does not necessarily reflect cysteine synthesis, as the cystathionase (CSE) enzyme that converts cystathionine to cysteine is not active in all cell types. Elevated cystathionine levels may therefore indicate a bottleneck in cysteine production. Similarly, measuring extracellular or intracellular cystine as a proxy for cysteine availability is problematic, as the conversion of cystine to cysteine is also largely dependent on the cellular NADPH/NADP^+^ ratio. Thus, cystine levels do not accurately reflect the amount of reduced cysteine available for cellular processes.

### 3.3 Recommendation

Given these limitations, direct detection of cysteine and glutathione using LC-MS with NEM derivatization is strongly recommended for accurate assessment of cysteine levels and cellular redox status.
